# Magnetic Resonance Imaging Findings in the Muscle Tissue of Patients with Limb Girdle Muscular Dystrophy Type 2I Harboring the Founder Mutation c.545A>G in the* FKRP* Gene

**DOI:** 10.1155/2018/3710814

**Published:** 2018-05-29

**Authors:** Zhiying Xie, Jiangxi Xiao, Yiming Zheng, Zhaoxia Wang, Yun Yuan

**Affiliations:** ^1^Department of Neurology, Peking University First Hospital, Beijing 100034, China; ^2^Department of Radiology, Peking University First Hospital, Beijing 100034, China

## Abstract

Limb girdle muscular dystrophy type 2I (LGMD2I) is an autosomal recessive muscular dystrophy that is rare in Asia and is caused by mutations in the fukutin-related protein gene (*FKRP*). The aim of this study was to determine if there are any characteristic features of muscle on magnetic resonance imaging (MRI) in patients with LGMD2I harboring the founder mutation c.545A>G in* FKRP*. Using MRI, we delineated changes in the thigh muscles of ten patients with genetically confirmed LGMD2I. The majority of muscle biopsy specimens showed reduced glycosylation of *α*-dystroglycan, decreased expression of laminin *α*2, and a dystrophic pattern. In our cohort, the muscles with the most severe fatty infiltration were adductor magnus and vastus intermedius, whereas the rectus femoris, sartorius, and gracilis muscles were relatively spared. In seven patients, we identified a concentric fatty infiltration pattern that was most pronounced in the vastus intermedius and vastus medialis muscles around the distal femoral diaphysis. In this disease, the initial fatty infiltration of the posterior thigh muscles gradually progresses anteriorly regardless of the founder mutation in* FKRP*. Muscle tissue in patients with LGMD2I who have the founder mutation c.545A>G in* FKRP* shows a distinctive concentric pattern of fatty infiltration and edema on MRI.

## 1. Introduction

Mutations in the fukutin-related protein gene (*FKRP*) have been shown to cause limb girdle muscular dystrophy type 2I (LGMD2I) [1, 2], which is common in Europe and North America but relatively rare in Asia. LGMD2I is an autosomal recessive muscular dystrophy characterized by progressive weakness and atrophy of the proximal muscles along with elevation of serum creatine kinase (CK) and frequently presents with cardiac and respiratory dysfunction [1, 3–5]. The phenotype of LGMD2I is heterogeneous, ranging from a mild form that manifests as asymptomatic hyperCKemia [6] or mild calf hypertrophy with onset in adulthood to a severe and early-onset form similar to Duchenne muscular dystrophy [7]. Muscle biopsies from patients with LGMD2I usually show myopathic changes with reduced glycosylation of *α*-dystroglycan (*α*-DG), which probably reflects pathogenic variations in the* FKRP* encoding a putative Golgi-resident glycosyltransferase [8, 9]. Currently, confirmatory diagnosis of LGMD2I is primarily dependent on genetic testing for* FKRP*.

It has been reported that there is no correlation between the clinical disease severity, histopathology, and glycosylated *α*-DG levels in patients with LGMD2I [10]. Muscle magnetic resonance imaging (MRI) is a noninvasive tool that can contribute to the diagnosis and assessment of disease severity and progression in a number of neuromuscular disorders. Several studies [3, 6, 11–14] have described the muscle MRI findings in European and North American patients with LGMD2I harboring the founder mutation c.826C>A in* FKRP*, which is different from the c.545A>G mutation found in mainland China [15]. Most of these studies [6, 11, 12, 14] suggest that there is initial fatty infiltration of the posterior thigh muscles with gradual progression anteriorly as the disease progresses. However, there have been some inconsistent findings in these studies, and the significance of muscle edema in LGMD2I is unknown. It has yet to be determined whether there are differences in the changes seen on MRI between patients with LGMD2I carrying the founder mutation c.545A>G and those carrying c.826C>A. In this study, we delineated the changes seen on muscle MRI, including fatty infiltration, edema, and abnormal muscle bulk, in patients with LGMD2I and the founder mutation c.545A>G in* FKRP*.

## 2. Materials and Methods

### 2.1. Patients

Ten unrelated patients (5 males, 5 females) with a genetically confirmed diagnosis of LGMD2I who presented to the Department of Neurology at Peking University First Hospital participated in the study. A detailed neurologic history was taken in all cases. Physical examinations were performed by two experienced neurologists who were blinded to the MRI findings. The main clinical characteristics and MRI findings for each patient are summarized in Table 1. The median patient age was 16 (range 3–37) years. The median age at onset was 9.5 (range 1.5–29) years and the median disease duration at the time of muscle MRI was 8.5 (range 1.5–20) years.

The physical examinations revealed that muscle strength measured using the Medical Research Council grading system [16] was lower than normal (proximal lower limb muscle strength in patients 2–9 ranged from 44% to 96%, normal 100%). All patients, with the exception of patient 1, who had an asymptomatic hyperCKemia phenotype, had a characteristic clinical phenotype that included proximal muscle weakness. One patient (patient 9) had a severe phenotype accompanied by marked muscle weakness in the distal limbs (60%). The phenotype was more severe in female patients than in male patients. In addition to proximal lower limb muscle weakness (found in 44%–88% of female patients and 88%–100% of male patients), there was weakness of the neck flexors in four female patients accompanied by weakness of the distal limbs in three female patients. The strength of the neck flexor and distal limb muscles was normal in the male patients. According to the ambulatory status scoring system proposed by Stensland et al. [17], eight patients had a score of 0 (walking without an aid) and two (patients 9 and 10) had a score of 2 (requiring a wheelchair at walking distances >200 m) at the time of muscle MRI examination. With the exception of patient 9, none of the patients had symptoms or signs of cardiomyopathy or respiratory insufficiency. Patient 9 had chest tightness, shortness of breath, and palpitation after physical activity, but had no echocardiographic abnormalities. Serum CK was markedly elevation in all patients (range 643–23131 IU/L, normal 25–170 IU/L). Three levels of disease severity (mild, moderate, and severe) were identified according to muscle strength and ambulation status.

Genetic testing showed that all patients carried the founder mutation c.545A>G in* FKRP*. Four patients were homozygous for the c.545A>G mutation and the remaining six patients were compound heterozygous for the c.545A>G mutation; the other heterozygous mutations were c.204_206delCTC, c.1263C>A, c.1067T>C, c.1027G>T, c.534G>T, and c.160C>T. The founder mutation c.545A>G and the other pathogenic variations in* FKRP* observed in this study have been reported previously [15, 18, 19].

The study was approved by the Ethics Committee of Peking University First Hospital. Written informed consent was obtained from all the study participants and/or their parents.

### 2.2. Muscle Biopsy

Muscle biopsies were obtained from quadriceps femoris (patient 7), deltoid (patient 8), or biceps brachii (normal control and patients 1–6, 9, and 10). The muscle specimens were frozen in isopentane, cooled in liquid nitrogen, and then stored at −80°C. Standard techniques were used for histochemical [20] and immunohistochemical [10] staining. Frozen sections (8-*μ*m thick) were processed for hematoxylin and eosin, NADH dehydrogenase, modified Gomori trichrome, Oil red O, Periodic acid-Schiff, ATPase, and nonspecific esterase staining. For immunohistochemistry analysis (normal control and patients 2, 4–8, and 10), the following primary antibodies were used: *α*-DG mouse monoclonal antibody, clone IIH6C4 (1:20, 40 *μ*l; no. 05-593; immunogen, rabbit skeletal muscle membrane preparation; Merck Millipore, Darmstadt, Germany), and laminin *α*2 mouse monoclonal antibody, clone 5H2 (1:20, 40 *μ*l; no. MAB1922; immunogen, purified human merosin; Merck Millipore). The muscle specimens from patients 1, 3, and 9 were not subjected to immunohistochemical staining because of an insufficient amount of tissue.

### 2.3. Muscle MRI

#### 2.3.1. Protocol

Muscle MRI examinations of both thighs were performed in the patients with LGMD2I using a 1.5-T MR scanner (GE 1.5 Sigma Twin Speed; GE Healthcare, Waukesha, WI, USA) with axial scanning in conventional T1-weighted and short T1 inversion recovery (STIR) sequences. The top part of the coil was at the level of the anterior superior iliac spine, and the scanning range covered the muscles of the pelvis to those of the thigh. Additional images were acquired in the coronal plane when necessary. The patients were asked to rest for at least half an hour before muscle MRI to avoid the effects of activity or exercise. The muscles were scanned in the noncontracted state. Patients 1 and 2 were routinely sedated to avoid movement artifacts. The parameters used for acquisition of the axial T1-weighted images were as follows: repetition time 625.0 ms, echo time 11.1 ms, matrix 320×256, slice thickness 5.0–8.0 mm, slice gap 0.5–7.5 mm, and field of view 28–32 cm. The parameters used for acquisition of the axial STIR images were as follows: repetition time 6225.0 ms, echo time 85.0 ms, matrix 320×256, slice thickness 5.0–8.0 mm, slice gap 0.5–7.5 mm, and field of view 28–32 cm. The total acquisition time for the axial T1-weighted and STIR images was approximately 20 minutes.

#### 2.3.2. MRI Interpretation

Fatty infiltration of muscle was graded on axial T1-weighted images using a modified 0–5-point Mercuri's scale (normal appearance to complete fatty infiltration) [21, 22] and muscle edema was graded on axial STIR images using the 0–5-point Stramare's scale (normal to moderate to global intrafascicular edema) [23]. The scans were also assessed for normal and abnormal muscle bulk (hypertrophy and atrophy) on axial T1-weighted images [11]. The MRI findings were independently interpreted by an experienced radiologist and a neurologist, who were blinded to all clinical information at the time of reviewing the images. Any disagreements were resolved by consensus. Thirteen individual muscles in both thighs were evaluated in each patient. The changes in the muscles seen on MRI were correlated with the type of mutation and the clinical characteristics of each patient.

### 2.4. Statistical Analysis

The Shapiro-Wilk test was used to confirm that the measured variables were not normally distributed. The median Mercuri and Stramare scores were calculated for the individual muscles to describe the pattern of muscle involvement. The median patient age, age at time of onset, and disease duration were treated as descriptive statistics. A two-tailed Spearman rank-order correlation coefficient (r_s_) was used to analyze the relationship between the main clinical characteristics (age, age at onset, disease duration, and muscle strength) indicating disease severity, type of mutation, and cumulative scores for fatty infiltration or edema in all the muscles evaluated. Positive and negative Spearman's correlations were considered statistically significant if the P value was <0.05. All statistical analyses were performed using SPSS for Windows version 22.0 (IBM Corp., Armonk, NY, USA).

## 3. Results

### 3.1. Muscle Pathology

**Figure 1:**
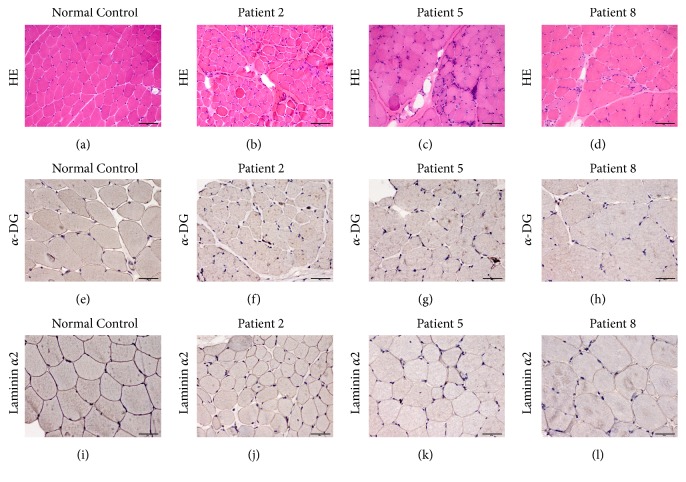
Pathologic changes in muscle in patients with limb girdle muscular dystrophy type 2I. (a, e, i) Normal control. (b, f, j) Patient 2. (c, g, k) Patient 5. (d, h, l) Patient 8. (a–d) Hematoxylin-eosin staining, HE (×20); (e–h) glycosylated *α*-DG immunohistochemistry staining (×40); (i–l) laminin *α*2 immunohistochemistry staining (×40). (b, c, d) Hematoxylin-eosin staining for patients 2, 5, and 8 showed dystrophic changes. (f, g, h) Immunolabeling for glycosylated *α*-DG in patients 2, 5, and 8 showed decreased *α*-DG glycosylation. (j, k, l) Immunolabeling showed mildly reduced laminin *α*2 expression in patients 2, 5, and 8.

The muscle biopsies for patients 1–3, 5, and 7–10 (n = 8, 80%) showed dystrophic changes, i.e., increased variation in fiber size, necrotic and regenerated fibers, and proliferation of connective tissue (Figures 1(b)–1(d)). The muscle biopsies for patients 4 and 6 showed only nonspecific myopathic changes, including a few hypertrophic, atrophic, and whorled fibers, as well as fiber splitting and a small number of internal nuclei. Muscle sections from patients 2, 4–8, and 10 (n = 7, 70%) immunolabeled for *α*-DG with IIH-6 showed decreased *α*-DG glycosylation (Figures 1(f)–1(h)). In the same 7 patients, immunolabeling revealed mildly reduced expression of laminin *α*2 (Figures 1(j)–1(l)).

### 3.2. Fatty Infiltration

The distribution and degree of fatty infiltration of the involved muscles was symmetric on both sides on axial T1-weighted images. The percentages of fatty infiltration with each score, along with the median score for each muscle, are shown in Table 2. The adductor magnus and vastus intermedius muscles were the most affected, with 70% showing severe fatty infiltration (scores 4 and 5). The adductor longus muscle had the next highest percentage of severe fatty infiltration, followed by gluteus maximus, vastus medialis, and the short and long heads of biceps femoris. The semitendinosus, semimembranosus, and vastus lateralis muscles were almost equally involved and showed mainly moderate fatty infiltration (scores 2 and 3). The rectus femoris, sartorius, and gracilis muscles were relatively spared and showed only mild fatty infiltration. The mean cumulative fatty infiltration score was 38.6 (standard deviation 5.9) in female patients and 25.4 (standard deviation 15.1) in male patients.

**Figure 2:**
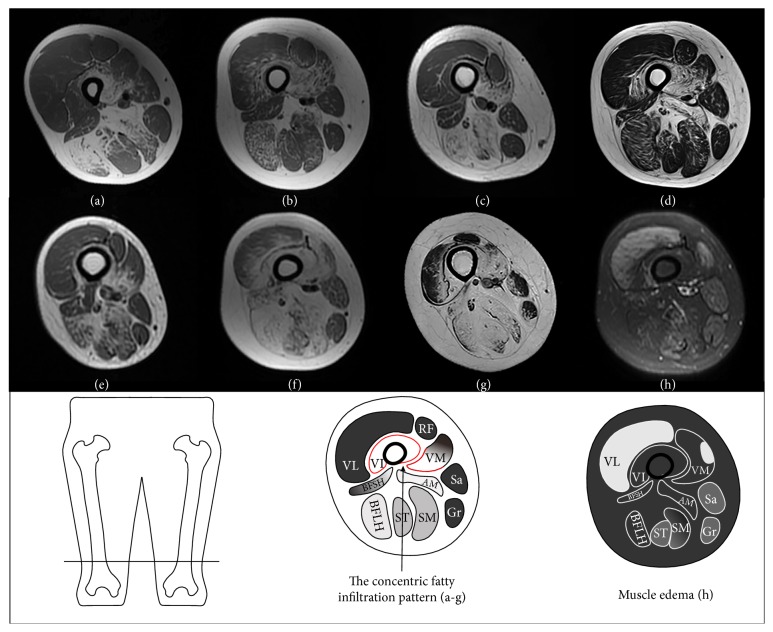
Axial T1-weighted images of the right thigh for patients 4–10 (a–g) showing a concentric fatty infiltration pattern around the distal femoral diaphysis and an axial short T1 inversion recovery image of the right thigh in patient 9 (h) showing relatively marked edematous changes in the thigh muscles.

Axial T1-weighted images at the level of the distal femoral diaphysis showed a concentric fatty infiltration pattern in patients 4–10 (Figures 2(a)–2(g)), consisting of severe fatty infiltration of vastus intermedius and the medial portion of vastus medialis, usually with relative sparing of vastus lateralis, rectus femoris, the lateral portion of vastus medialis, and the short head of biceps femoris. The concentric fatty infiltration pattern was not observed in patients 1–3, who had mild disease severity (Table 1).

**Figure 3:**
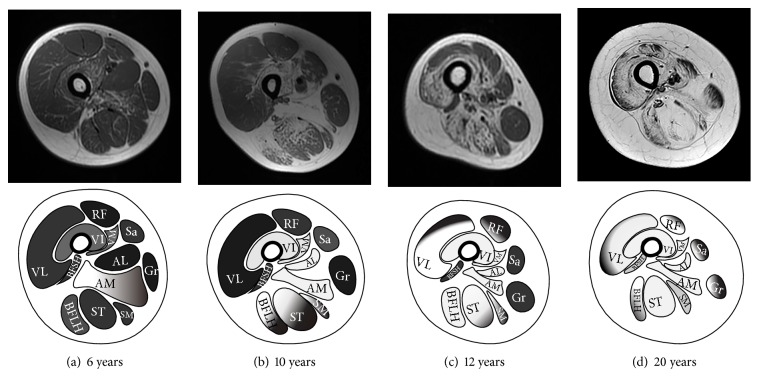
Axial T1-weighted images of the right thigh for (a) patient 3, (b) patient 4, (c) patient 8, and (d) patient 10 at the level of the mid-femoral diaphysis showing initial fatty infiltration of the posterior thigh muscles with gradual progression anteriorly as the disease progressed.

Axial T1-weighted images at the level of the mid-femoral diaphysis showed that fatty infiltration was related to the duration and severity of disease (Figure 3). In patient 3 (Figure 3(a)), who had mild disease severity and a disease duration of 6 years, the fatty infiltration was only apparent in the adductor magnus muscle with slight involvement of vastus intermedius. In patient 4 (Figure 3(b)), who had moderate disease severity and a disease duration of 10 years, there was obvious fatty infiltration in the vastus intermedius and vastus medialis muscles in addition to severe fatty infiltration of adductor magnus, adductor longus, and the long head of biceps femoris. Compared with patient 4, in patient 8 (Figure 3(c)), who had a disease duration of 12 years, the fatty infiltration was more severe in the posterior thigh muscles than in the anterior thigh muscles. In patient 10 (Figure 3(d)), who had a disease duration of 20 years and a severe phenotype, the posterior and anterior thigh muscles were almost equally affected by fatty infiltration, representing end-stage appearance of the disease.

### 3.3. Muscle Edema

On axial STIR images, the distribution and degree of muscle edema of the involved muscles were symmetric on both sides. The percentages of muscle edema with each score, along with the median score for each muscle, are shown in Table 2. The adductor longus muscle was the most affected, with 40% showing slight muscle edema. Gluteus maximus, rectus femoris, vastus lateralis, and the long head of biceps femoris were equally affected, with 30% showing slight muscle edema. The remaining thigh muscles showed very slight or no muscle edema. Patient 9 (Figure 2(h)) showed relatively marked edematous changes in the thigh muscles with a cumulative muscle edema score of 20, while the other patients showed very slight or no muscle edema (Table 1).

### 3.4. Abnormal Muscle Bulk

Hypertrophy was found in the muscles with slight fatty infiltration. The gracilis and sartorius muscles were hypertrophic in 60% and 50% of the patients, respectively. Hypertrophy of the rectus femoris, vastus lateralis, and semitendinosus muscles was present in 30% of patients. Eighty percent of the patients had prominent atrophy of the adductor magnus muscle with severe fatty infiltration. There was no obvious hypertrophy or atrophy in the remaining thigh muscles (Table 2).

### 3.5. Correlations

There was a significant positive correlation of the cumulative fatty infiltration scores for all muscles evaluated with disease duration (r_s_ = 0.924,* P* < 0.01). There was also a significant negative correlation of the cumulative fatty infiltration scores with proximal muscle strength in the lower limbs (r_s_ = −0.920,* P* < 0.01) and upper limbs (r_s_ = −0.937,* P* < 0.01), as well as with neck flexors strength (r_s_ = −0.680,* P* < 0.05). However, no statistically significant correlation was found between the cumulative scores for muscle edema and clinical characteristics (sex, age at onset, disease duration, and muscle strength). The type of mutation (homozygous or heterozygous) did not correlate with muscle strength or the cumulative scores for fatty infiltration or muscle edema.

## 4. Discussion

In our series from China, all patients with LGMD2I had proximal muscle weakness, with the exception of one patient who had asymptomatic hyperCKemia. All of these patients harbored the founder mutation c.545A>G in* FKRP*. A previous study indicated that Chinese patients who were homozygous for the c.545A>G mutation had a less severe phenotype than those with compound heterozygous mutations [15]. However, we found no significant difference in disease severity between patients who were homozygous for the c.545A>G mutation and those with compound heterozygous mutations.

Nonspecific myopathic changes have been reported to be common in patients with the c.826C>A mutation [10]. However, in this series of Chinese patients with LGMD2I and the c.545A>G mutation, the majority of muscle biopsies showed the dystrophic pattern. Correct glycosylation of *α*-DG, a component of the dystrophin-associated glycoprotein complex, is crucial for binding of laminin *α*2, so hypoglycosylation of *α*-DG as a result of mutations in* FKRP* would inevitably influence the interaction with laminin *α*2 [2, 8, 10, 24]. Our muscle pathology findings, including reduced glycosylation of *α*-DG and decreased laminin *α*2 expression on immunohistochemistry staining, further indicate that hypoglycosylation of *α*-DG is related to loss of interaction with laminin *α*2 in LGMD2I, which in turn leads to depletion of laminin *α*2 [10].

This is the first report on muscle MRI changes, including fatty infiltration, edema, and abnormal muscle bulk in Chinese patients with LGMD2I and the founder mutation c.545A>G in* FKRP*. In our cohort, the muscles most severely affected by fatty infiltration in patients with the c.545A>G mutation were adductor magnus and vastus intermedius, followed by adductor longus, gluteus maximus, vastus medialis, and biceps femoris, whereas the muscle most severely affected in patients with the c.826C>A mutation was the long head of biceps femoris [6] or adductor magnus [11, 13]. In the present study, we found that fatty infiltration of the gluteus maximus [3, 6, 12, 14], semimembranosus [6, 11, 12], and semitendinosus [6, 12, 14] muscles was not as severe as reported in patients with the founder mutation c.826C>A or the common mutation c.948delC [25] in* FKRP*. Willis et al. [6] identified a gender-related difference in patients with the c.826C>A mutation, whereby the median value for fat infiltration in vastus medialis was greater than that in vastus lateralis in male patients but not in female patients. However, in our study, we found that fatty infiltration was more severe in female patients than in male patients, which was consistent with a gender-related difference in disease severity. Relative sparing and hypertrophy of the rectus femoris, sartorius, and gracilis muscles appeared to be a frequent finding in patients with the c.545A>G mutation, which is consistent with previous reports in patients with the c.826C>A mutation [3, 6, 11, 12]. As in the previous studies of patients with the c.826C>A mutation [6, 11, 12, 14], our study also demonstrated that initial fatty infiltration of the posterior thigh muscles with gradual progression anteriorly as the disease progressed in patients with the c.545A>G mutation. The pattern of fatty infiltration in predominantly the posterior thigh muscles, especially adductor magnus and the hamstrings, is similar to that in* POMT*-related a-dystroglycanopathy, indicating that the a-dystroglycanopathies, including LGMD2I, may present with a distinctive pattern of muscle involvement [26].

The concentric fatty infiltration pattern around the distal femoral diaphysis was identified first in most of our patients and appears to be a distinctive pattern in LGMD2I with the c.545A>G mutation, but was only observed in patients with the c.826C>A mutation in the studies reported by Willis et al. [6, 12]. The concentric fatty infiltration pattern is reportedly rare in other neuromuscular disorders, including alpha-sarcoglycanopathy [11], dystrophinopathy with a clinical phenotype overlapped with LGMD2I [11, 27], collagen VI-related myopathy [28], laminopathies [29],* RYR1*-related myopathies [30], congenital muscular dystrophy with rigid spine syndrome [31, 32], Emery-Dreifuss muscular dystrophy caused by mutations in* LMNA* [33, 34], dysferlinopathy [35], neutral lipid storage disease with myopathy caused by mutations in* PNPLA2* [36], and inflammatory myopathy with anti-SRP antibodies [37]. Nevertheless, this pattern could be useful in the diagnosis of LGMD2I and guide genetic testing for* FKRP*.

It was reported that quantitative fat imaging using the Dixon technique could monitor progression of the disease over a 12-month period whereas conventional T1-weighted imaging could not [12]. However, quantitative fat imaging with the Dixon technique has not been routinely applied in clinical practice because it is time-consuming, challenging in terms of expertise, and more technically demanding than T1-weighted imaging [38]. In our study, there was a significant correlation of the extent of fatty infiltration, as assessed by Mercuri scoring of T1-weighted images, with disease duration and muscle strength. Furthermore, it has been reported that histopathologic alterations, levels of *α*-DG hypoglycosylation, and laminin *α*2 depletion are not suitable markers of the clinical severity of LGMD2I [10]. Thus, the fatty infiltration assessed by using the Mercuri's scale could be used as an objective marker for evaluating the severity of LGMD2I. A longer-term longitudinal study is needed to determine if fatty infiltration assessed by Mercuri's scale would be a more objective marker of the severity and progression of LGMD2I than physical function testing using the Medical Research Council muscle strength grading system and time to rise from the floor, which relies heavily on the effort and motivation of the patient.

It is known that fatty infiltration of muscles is an irreversible pathologic change in myopathy, whereas muscle edema is potentially reversible. Although there are still no published studies on muscle edema in LGMD2I, its importance should not be underestimated. However, in view of the common finding of very slight or no muscle edema in most of our patients, muscle edema would not be a common imaging finding in LGMD2I. However, we cannot explain why one of our patients showed relatively marked edematous changes in the thigh muscles. We found no correlation between the degree of muscle edema and clinical characteristics or the type of mutation. Additionally, the thigh muscle that showed the most edema was adductor longus muscle, which was not the muscle showing the most severe fatty infiltration. Therefore, muscle edema is unlikely to be specific for LGMD2I, contribute to muscle weakness, or assist in evaluation of the functional status of patients with the disease.

In summary, in patients with LGMD2I, there is initial fatty infiltration of the posterior thigh muscles with gradual progression anteriorly regardless of the type of founder mutation in* FKRP*. This study highlights a distinctive pattern of muscle involvement, i.e., concentric fatty infiltration, in LGMD2I with the founder mutation c.545A>G in* FKRP*. Our findings also indicate that muscle MRI could be used as an objective tool for evaluation of disease severity and furthering our understanding of the natural history of LGMD2I.

## Figures and Tables

**Table 1:** Clinical characteristics and findings on magnetic resonance imaging of muscle in patients with limb girdle muscular dystrophy type 2I.

Patient	Age, years /sex	*FKRP* mutation	Age at onset, years	Disease duration, years	Muscle strength*∗* (%)					Ambulatory status^#^	Disease severity	Cumulative score		Con
					Neck flexors	UL Proximal	UL Distal	LL Proximal	LL Distal			Fatty infiltration	Muscle edema	
1	3/M	c.545A>G c.204_206delCTC	1.5	1.5	100	100	100	100	100	0	0	0	0	-
2	4/M	c.545A>G c.1263C>A	2	2	100	100	100	96	100	0	0	25	2	-
3	14/M	c.545A>G hom.	8	6	100	100	100	92	100	0	0	29	13	-
4	13/M	c.545A>G c.1067T>C	3	10	100	90	100	88	100	0	1	34	2	+
5	34/M	c.545A>G hom.	14	20	100	90	100	88	100	0	1	39	1	+
6	36/F	c.545A>G hom.	29	7	100	100	80	88	100	0	1	33	5	+
7	16/F	c.545A>G c.1027G>T	11	5	80	100	100	84	100	0	1	33	2	+
8	24/F	c.545A>G hom.	12	12	40	80	100	72	100	0	2	40	0	+
9	16/F	c.545A>G c.534G>T	2	14	80	80	60	44	60	2	2	40	20	+
10	37/F	c.545A>G c.160C>T	17	20	80	60	80	44	100	2	2	47	0	+

*∗*, muscle strength was converted using the Medical Research Council formula [16]; #, ambulatory status was scored as follows [17]: walking without aid (0), walking with an aid (1) (e.g., stick, crutch, or frame), need for a wheelchair at walking distances >200 m (2), and wheelchair dependence (3). Disease severity: mild (0), moderate (1), and severe (2); -, the patient did not present with a concentric fatty infiltration pattern; +, the patient presented with a concentric fatty infiltration pattern. Abbreviations: Con, concentric fatty infiltration pattern; F, female; hom., homozygous; LL, lower limbs; M, male; UL, upper limbs

**Table 2:** Percentages of fatty infiltration, muscle edema scores, abnormal muscle bulk for the individual thigh muscles, and median scores for each muscle.

Muscle	Fatty infiltration score							Muscle edema score							Abnormal muscle bulk	
	0	1	2	3	4	5	Median	0	1	2	3	4	5	Median	Hypertrophy	Atrophy
GM	10	0	30	20	40	0	3	70	10	20	0	0	0	0	NE	10
RF	10	20	50	20	0	0	2	70	10	20	0	0	0	0	30	NE
VL	10	0	50	30	10	0	2	70	10	20	0	0	0	0	30	10
VI	10	0	20	0	70	0	4	90	0	10	0	0	0	0	10	10
VM	10	0	10	50	30	0	3	80	10	10	0	0	0	0	20	10
Sa	10	20	70	0	0	0	2	80	20	0	0	0	0	0	50	NE
AL	20	0	30	0	50	0	3	60	10	30	0	0	0	0	20	30
AM	10	10	0	10	70	0	4	80	0	20	0	0	0	0	NE	80
Gr	10	30	50	10	0	0	2	100	0	0	0	0	0	0	60	NE
SM	10	0	50	20	20	0	2	90	0	10	0	0	0	0	NE	20
ST	10	0	50	20	20	0	2	80	10	10	0	0	0	0	30	10
BFLH	10	0	40	10	30	0	2.5	70	10	20	0	0	0	0	NE	10
BFSH	10	10	30	20	30	0	2.5	90	0	10	0	0	0	0	NE	NE

AL, adductor longus; AM, adductor magnus; BFLH, biceps femoris, long head; BFSH, biceps femoris, short head; GM, gluteus maximus; Gr, gracilis; NE, nonexistent; RF, rectus femoris; Sa, sartorius; SM, semimembranosus; ST, semitendinosus; VL, vastus lateralis; VI, vastus intermedius; VM, vastus medialis
